# The consequences of the new European reclassification of non-invasive brain stimulation devices and the medical device regulations pose an existential threat to research and treatment: An invited opinion paper ☆

**DOI:** 10.1016/j.clinph.2024.03.039

**Published:** 2024-04-10

**Authors:** Andrea Antal, Ana Ganho-Ávila, Sara Assecondi, Tracy Barbour, Jovana Bjekić, Daniel M. Blumberger, Nadia Bolognini, Jerome Brunelin, Lorena Chanes, Matthew Dale, Raffaele Dubbioso, Giordano D’Urso, Igor Filipcic, Saša R. Filipović, Marco Hirnstein, Femke Konings, Berthold Langguth, Letizia Leocani, Majid Memarian Sorkhabi, Marc Mulder, Mika Nikander, Rafal Nowak, Antonio Oliviero, Balder Onarheim, Jacinta O’Shea, Stefano Pallanti, Fady Rachid, Joana Rajão-Saraiva, Simone Rossi, Alexander T. Sack, Anne Sauvaget, Rik van der Scheer, Klaus Schellhorn, Aureli Soria-Frisch, David Szekely, Hatice Tankisi, Paul CJ. Taylor, Indira Tendolkar, Susanne Uusitalo, Chris Baeken

**Affiliations:** a Department of Neurology, University Medical Center Göttingen, Göttingen. Germany; b Center for Research in Neuropsychology and Cognitive Behavioral Intervention, Faculty of Psychology and Educational Sciences, University of Coimbra, Coimbra, Portugal; c Centre for Mind/Brain Sciences - CIMeC, University of Trento, Rovereto (TN), Italy; d Massachusetts General Hospital, Department of Psychiatry, Harvard Medical School, Boston, MA, USA; e University of Belgrade, Institute for Medical Research, Human Neuroscience Group and Centre for Neuroscience and Neuromodulation Belgrade, Serbia; f Temerty Centre for Therapeutic Brain Intervention, Centre for Addiction and Mental Health and Department of Psychiatry, Temerty Faculty of Medicine, University of Toronto, Toronto, ON, Canada; g Department of Psychology, University of Milano Bicocca, and Laboratory of Neuropsychology, IRCCS Istituto Auxologico Italiano, Milano, Italy; h CH Le Vinatier, Université Claude Bernard Lyon 1, CNRS, INSERM, Centre de Recherche en Neurosciences de Lyon CRNL, Bron, France; i Department of Clinical and Health Psychology-Institute of Neurosciences, Universitat Autònoma de Barcelona, Barcelone, Spain; j Magstim, Spring Gardens, Whitland, Carmarthenshire, SA34 0HR, UK; k Neurophysiology Unit, Department of Neurosciences, Reproductive Sciences and Odontostomatology, University of Naples, Naples, Italy; l Department of Neurosciences, Reproductive and Odontostomatological Sciences, University of Naples “Federico II”, Naples, Italy; m Psychiatric Clinic ‘Sveti Ivan’, Zagreb, Croatia; n University of Belgrade, Institute for Medical Research, Human Neuroscience Group and Centre for Neuroscience and Neuromodulation, Belgrade, Serbia; o Department of Biological and Medical Psychology, University of Bergen, Bergen, Norway; p Independent expert by experience contributor, Amsterdam, the Netherlands; q Chair of the German Society for Brain Stimulation in Psychiatry, Department of Psychiatry and Psychotherapy, Bezirksklinikum, University of Regensburg, Germany; r Experimental Neurophysiology Unit, Institute of Experimental Neurology-INSPE, San Raffaele Scientific Institute, Milan, Italy; s Faculty of Medicine, Vita-Salute San Raffaele University, Milan, Italy; t Magstim, Spring Gardens, Whitland, Carmarthenshire SA34 0HR, UK; u Independent expert by experience contributor, The Hague the Netherlands; v Sooma Oy, Helsinki, Finland; w Neuroelectrics, Barcelona, Catalonia, Spain; x FENNSI Group, Hospital Nacional de PArapléjicos, SESCAM, Toledo, Spain; y Center for Clinical Neuroscience - Hospital “Los Madroños”, Brunete (Madrid), Spain; z School of Psychology and Humanities, University of Central, Lancashire, U.K; aa Department of Psychiatry, University of Oxford, U.K; ab Istituto di Neuroscienze (Italy) and Albert Einstein College of Medicine (NY. USA) Chair of ECNP Network on Neuromodulation; ac Private Practice, 7, place de la Fusterie, 1204, Geneva, Switzerland; ad Independent expert by experience contributor, Coimbra, Portugal; ae Siena Brain Investigation and Neuromodulation Lab (SiBIN Lab), Department of Medicine, Surgery and Neuroscience, University of Siena, Italy; af Section Brain Stimulation and Cognition, Department of Cognitive Neuroscience, Faculty of Psychology and Neuroscience, Maastricht University (UM); ag Department of Psychiatry and Neuropsychology, School for Mental Health and Neuroscience (MHeNs), Brain+Nerve Centre, Maastricht University Medical Center (MUMC+), Center for Integrative Neuroscience (CIN), the Netherlands; ah Department of Psychiatry, University Hospital of Nantes, France; ai Independent Patient Representative Advisor in Adult, Child & Adolescent Psychiatry, Venlo, the Netherlands; aj neuroConn GmbH, Ilmenau, Germany; ak Neuroscience BU, Starlab Barcelona, Spain; al Deputy Head of Neuromodulation Unit of Princess Grace Hospital Centre, Monaco; am Head of the Europa, Middle East, Africa Chapter of the International Federation of Clinical Neurophysiology, Department of Clinical Neurophysiology, Aarhus University Hospital and Department of Clinical Institute, Aarhus University, Aarhus, Denmark; an Department of Psychology, LMU Munich, Munich, Germany; ao Donders Institute for Brain, Cognition and Behavior, Department of Psychiatry, Radboud University Nijmegen, Netherlands; ap Philosophy (ethics), University of Oulu, Finland; aq Department of Head and Skin - Psychiatry and Medical Psychology, Ghent Experimental Psychiatry (GHEP) Lab, Ghent University, Ghent, Belgium; ar Vrije Universiteit Brussel (VUB), Universitair Ziekenhuis Brussel (UZ Brussel), Department of Psychiatry, Brussels, Belgium; as Department of Electrical Engineering, Eindhoven University of Technology, Eindhoven, the Netherlands

**Keywords:** Medical device regulation, Brain stimulation, Reclassification

## Abstract

A significant amount of European basic and clinical neuroscience research includes the use of transcranial magnetic stimulation (TMS) and low intensity transcranial electrical stimulation (tES), mainly transcranial direct current stimulation (tDCS). Two recent changes in the EU regulations, the introduction of the Medical Device Regulation (MDR) (2017/745) and the Annex XVI have caused significant problems and confusions in the brain stimulation field. The negative consequences of the MDR for non-invasive brain stimulation (NIBS) have been largely overlooked and until today, have not been consequently addressed by National Competent Authorities, local ethical committees, politicians and by the scientific communities. In addition, a rushed bureaucratic decision led to seemingly wrong classification of NIBS products without an intended medical purpose into the same risk group III as invasive stimulators.

Overregulation is detrimental for any research and for future developments, therefore researchers, clinicians, industry, patient representatives and an ethicist were invited to contribute to this document with the aim of starting a constructive dialogue and enacting positive changes in the regulatory environment.

## 1 Background

### 1.1 Introduction

Non-invasive brain stimulation (NIBS) methods, including transcranial magnetic stimulation (TMS) and low intensity transcranial electrical stimulation (tES, mainly transcranial direct current stimulation – tDCS) entered the international scientific and clinical community four decades ago (Lefaucheur et al. 2017, 2020; Rossi et al., 2009, 2021). European researchers, clinicians and manufacturers of NIBS devices have been pioneering the development of innovative NIBS technologies, research paradigms and clinical protocols to advance understanding of brain function in health, in neurological and psychiatric illnesses and beyond. All stakeholders have – from the beginning – shown great enthusiasm for NIBS approaches, recognizing their potential benefits for both neuroscientific research and clinical use while critically evaluating their safety. However, despite its pioneering role, numerous research and clinical NIBS activities in Europe face significant challenges today. Patient access to NIBS, supported by adequate reimbursement strategies in many EU countries, remains limited, even though efforts have been made over the past decade to introduce numerous quality and safety checks within the NIBS field. While standard operating procedures (SOPs) and related regulations are certainly necessary to reach further harmonization across Europe, this movement has introduced regulatory burdens for all of the stakeholders. Above all, two recent changes, the introduction of the Medical Device Regulation (MDR) (2017/745) and the Annex XVI have caused significant problems (Baeken et al., 2023; Bublitz, 2023).

Under the MDR, when conducting a clinical or research study with a medical device, outside of its intended purpose, a one-step approval from the local ethics committee cannot be provided for the planned research activities, and the registration of the study by the National Competent Authorities will be required (according to MDR Article 82). Not only does this process place a considerable burden on National Authorities and Ethical Committees, but there are also considerable variations in the way it is nationally handled.

More recently, another issue of concern has emerged with the reclassification of NIBS technologies (C/2022/8638; hereafter referred to as “Reclassification”). It is a blanket regulation, dictating a given risk class for a wide spectrum of technologies (see detailed explanations below). Here, transcranial stimulation products without an intended medical purpose were replaced from Class I to Class III.

Our understanding is that the Reclassification and the current legal situation in Europe results from inadequate and unrepresentative involvement of a very limited number of stakeholders during the development of the new regulation and the approval process. Therefore, our aim in this position paper is to give a voice to a wider representation of stakeholders in the NIBS field, mainly in Europe but also beyond, on the impact of this recent regulatory change, as well as to present and discuss the serious concerns – and possible solutions – arising from the interpretations related to MDR and the Reclassification of NIBS. Researchers, clinicians, industry and patient representatives and an ethicist (who is as a researcher and a member of a national medical research ethics committee and also involved in national appraisals for health technologies in the public health sector) were invited to contribute to this document with the aim of starting a constructive dialogue and enacting positive changes in the regulatory environment.

### 1.2 The medical device regulation (MDR)

The EU MDR (2017/745) came into force on 26 May 2021 (https://eumdr.com/). According to the original plans, the application of MDR should be implemented gradually through a so-called ‘transition period’ (Article 120) until 2028 (first plan was until 2024), meaning that existing NIBS products would be allowed to stay on the market until the end of this period, if these devices comply with a set of transition rules from Article 120. Such devices were classified as Class I and Class IIa according to the previous legal/regulatory framework, the Medical Device Directive (MDD). A *Regulation (new)*, as opposed to a *Directive (old)*, is a binding leg-islative act that must be applied in its entirety and in an identical manner across the EU Member States. The aim of the harmonization at the European level was to provide greater certainty, predictability, and transparency for using medical devices in research and, as a result, conducting clinical investigations, with the highest standards of research participants and patients’ safety, while also fostering collaborations between EU member states.^1^ Accordingly, the new MDR contains greater detail than the old Directive.

The different interpretations of the MDR (2017/745) by (mainly local or national) regulatory authorities have already hindered basic, translational, and clinical research from being carried out, not only those pertaining to the brain but also to peripheral nerve and muscle. In fact, many studies fall into the category of ‘*other clinical studies*’ as stated in the MDR. For example, such studies may propose to use exactly the same certified stimulators that are registered medical devices, with very similar participant and/ or patient populations, but outside the intended purpose, with the use of a different control electrode position or a slightly different stimulation duration. Under the MDR, these can no longer be authorized by permission of a local ethics committee but require registration with the National Competent Authority.

During the process of writing this article, some guidance documents were developed by the Medical Device Coordination Group (MDCG). Those documents are not legally binding. They present a common understanding of how the MDR should be applied in practice aiming at an effective and harmonized implementation of the legislation. However, the term “research” is not mentioned in any of those documents (see^2^).

The sudden tightening of the regulations and the lack of necessary clarifications have also slowed down research and innovation as well as the exploitation of scientific outputs. Furthermore, based on the Reclassification arguments put forward in C/2022/8638, there is a risk that even devices with intended medical purpose will end up in a higher class as (new) medical devices are being re-evaluated. In extreme cases, ethical committees outside medical institutions (e.g., in psychology faculties) are already unwilling to accept such projects, leading to the real risk that basic research in the field will cease.

## 2 The new EU regulation

### 2.1 Reclassification of NIBS devices without intended medical purpose

In December 2022, the EU reclassified repetitive transcranial magnetic stimulation (rTMS) and low intensity transcranial electrical stimulation (tES) without an intended medical purpose “*intended for brain stimulation that apply electrical currents or magnetic or electromagnetic fields that penetrate the cranium to modify neural activity in the brain*”, as Class III devices, i.e., the category of highest risk, a category, for example, which also comprises invasive neurosurgical procedures such as deep-brain stimulation.^3^ Under the previous regulatory framework (MDD), NIBS devices were mostly classified as active medical devices of Class IIa (manageable risks, approved treatment effects) and the NIBS devices with medical purpose stayed in this category. The Reclassification refers only to “*products without an intended medical purpose*”, raising two issues. First, it is unclear, which devices fall under label e.g. if it covers or not the cognitive performance enhancement devices, as well as supposedly research-only devices that are on the market (for further discussion of “intended purpose” see section 2.3 below). Second, the report on the risks and adverse effects of rTMS and tES on which the Reclassification process is based is substantially inaccurate and contradicts scientific evidence (see below). In summary, the Reclassification does not seem to have considered the high-quality data on NIBS safety gathered over the last decades. Based on the available evidence on adverse events (AE) and serious AEs (SAEs) (or with another terminology, incidents and serious incidents), only a limited number of SAEs were registered (Rossi et al, 2021, Antal et al., 2017). The above-mentioned concerns and the consequences of the Reclassification have motivated the European Society of Brain Stimulation (ESBS) (https://www.brain-stimulation.eu) to advocate against the content of the NIBS reclassification, as explained in their published “Manifesto” and an Editorial in the journal Brain Stimulation (Baeken et al., 2023, see also Bublitz, 2023).

How the Reclassification ruling was reached is hard to fathom. According to the explanation given for the ruling in July 2022, “*certain EU Member States jointly requested the reclassification of several active products without an intended medical purpose*” (Annex XVI), including NIBS devices. These EU member states were Spain, Portugal, France, Belgium, Luxembourg and the Netherlands. Supporting this request, the following explanation was given: “*As these products have no intended medical purpose, they have not been covered by the European Directive 93/42/EEC regarding medical devices and they were not submitted to the Materiovigilance system put in place for medical devices. Therefore, the national database on incidents related to the use of medical devices are not supposed to contain incidents related to the use of these Annex XVI products. However, we have sometimes received incident reports. Most of them concern the use of equipment emitting high-intensity electromagnetic radiation and resulting in burns*.”

Additionally, the following AEs are mentioned in their explanation: “*According to the literature: The risks linked to the use of equipment intended for brain stimulation are: atypical brain development, abnormal patterns of brain activity, increased metabolic consumption, fatigue, anxiety, irritability, headaches, muscle twitches, tics, seizures, vertigo, skin irritation at the electrode site*.”

As evidence for this claim, the rapporteur cited the following five papers from the literature (this part is copied out from the original letter written by the representatives of 6 member states, the letter was addressed to Ms Stella Kyriakides, Ref: Ares (2022) 5472220-29/97/2022):

6. Regulation of cognitive enhancement devices, Maslen et al, J. Law Biosci 2014
7. Mind Machines, Oxford Marin Policy Paper
8. Induction of Late LTP-Like Plasticity in the Human Motor Cortex by Repeated Non-Invasive Brain Stimulation, Monte-Silva et al, Brain Stimulation 6 (2013)
9. Novel neurotechnologies: intervene in the brain, Nuffield Council on Bioethics
10. Would you be willing to zap your child’s brain? Public perspectives on parental responsibilities and the ethics of enhancing children with transcranial direct current stimulation, Wagner et al, AJOB Empiral Bioethics, 2018

The claims that TMS/tES can induce “*atypical brain development*” or “*abnormal patterns of brain activity*” are wrong, totally misleading and not based on scientific evidence. In fact, those are quotes from almost ten-year-old opinion papers in which authors speculated about this possibility without relying on actual data. After 30 years of NIBS, there is still no data showing such adverse effects. Moreover, the claims about rTMS/tES-related seizure risks completely contradict the clinical and research data we have available in the literature for the last decades, which demonstrated that observed seizure rates in rTMS are much lower than previous guidelines suggested (Rossi et al., 2009; 2021, Caulfield et al. 2022), and in the context of tES, there have been no documented cases of stimulation-induced seizures (Antal et al. 2017, Bikson et al. 2018).

In fact, none of the five papers cited provide any empirical evidence to support the claimed risks, other than eventual transitory skin redness under the electrode. Four of the five papers are opinion pieces. Only one is an experimental study, and this one seems to be low relevance as it has no focus or data on safety. Rather, it reports measurements of motor evoked potentials in healthy subjects before and after different doses of tDCS. Considering that the five papers cited to justify the risks do not deal with safety data, we question on what basis these risks are claimed. We note that the first three stated risks (among the most concerning) are strikingly similar in wording to a paragraph from another theoretical opinion paper, which was not cited: Cohen Kadosh et al (2012), Current Biology, Vol 22, Nr 4, page R109. *“For example, repeated stimulation of the parietal cortex in order to increase numerical competence during developmental stages when the prefrontal cortex is more important [13] might not only fail to give any improvement but it could even worsen performance and lead to atypical brain development. Like other types of atypical experience during sensitive periods [14], the stimulation of the wrong brain area might induce abnormal patterns of brain activity in this brain region and interconnected areas, and increase metabolic consumption in brain areas that are irrelevant to the specific psychological function*.” Importantly, this essay is speculating (“might”, “could”) about potential adverse impacts of hypothetical future stimulation protocols if safety issues are not carefully followed. The essay calls for “*more research into the safety and potential hazards*” so that risk assessments can be evidence-based. It would be ironic, indeed, if this theoretical speculation and call for evidence-based risk assessments were grossly misconstrued as actual empirical evidence of real harm. Especially considering the 10 years of safety studies since, clearly documenting that no such effect can be found.

Turning to the actual evidence base for NIBS safety, there is significant literature that has not been addressed. We conducted a search in the PubMed scientific database (September 1, 2023) for just one of the NIBS methods, screening for papers with “tDCS” or “transcranial direct current stimulation” as a keyword. This resulted in a total of 9588 publications, about 40 % of these papers mention safety of methods and describe side and adverse effects during the applications or report the absence of the effects. Five papers were cited by the EU member states (see above). tDCS represents only one of the (at least) twelve established NIBS technologies we are aware of as being impacted by the Reclassification. Thus, in their selection of references, the last years of evidence, including the results of recent guidelines, opinion papers and *meta*-analyses were ignored.

As for another NIBS method, rTMS, in 2021 an international scientific consortium consisting of 37 international experts reviewed again (a first revision had been carried out in 2009) the entire available data on TMS safety in adults and children and updated their published recommendations on the safety for TMS/rTMS use (Rossi et al., 2021). The presented data suggest such extremely low seizure risks that some of the previous safety concerns (Rossi et al., 2009) are no longer supported. With now TMS approaching 40 years of steady increasing usage and declining reports and concerns about safety, the available scientific and clinical data is in complete contradiction to the argument underlying the Reclassification. The same is true for low intensity tES, which have consistently shown minimal safety concerns (Bikson et al., 2023).

However, the representatives of the countries summarize their worries in the following way in the above-mentioned joint letter: “*We believe that there is enough scientific evidence to clearly establish the risks for these groups of products. Moreover, as these products have no intended medical purpose, their use can’t induce any risk for the public nor for the professional user. In application of the precautionary principle, defined by the EU Court of Justice as a general principle of Community law requiring the competent authorities to take appropriate measures to prevent specific potential risks to public health, safety and the environment, by giving precedence to the requirements related to the protection of those interests over economic interests, we should not wait until the reality and seriousness of those risks become fully apparent before reclassifying these products*.”

The public consultation process on Reclassification involved an EU group called SANTE (Directorate-general for Health and Food Safety) preparing a draft, which was published for an 8-week hearing period on the *Have Your Say* platform. To the best of our knowledge, no experts in the field or NIBS’ companies were notified about this process or hearing period. The draft received only 22 comments from the public and almost all of them were related to non-NIBS devices. On December 1st, 2022 the final version of the Reclassification was published and it became effective in law from 22nd December onwards, meaning that, as of today, all new non-medical NIBS products in the EU have to comply with Class III rules.^4^

Only nine working days elapsed between the EU Commission’s receipt of the Member States’ letter and the publication of the Reclassification for consultation. It is hard to imagine that a major investigation into such an unexpected finding could be completed in just a few days. When the EU Commission was asked to provide the scientific references that supported the Reclassification, they shared the list from the member states’ letter. This list contained the same inaccuracies as the previous document, which might suggest a lack of thoroughness of the internal process, aimed to be independent of the member states’ letter.

### 2.2 New requests when planning the basic and clinical studies to comply with new regulation

The first important question is whether NIBS devices are medical or non-medical devices. Problems may arise when the categorical distinction between the two is unclear, or when a given stimulator has both medical and non-medical purposes (e.g., improving cognition).

Even when the stimulator is a medical device, in order to determine the regulatory pathway for studies with CE-marked NIBS devices, it is necessary to understand the *intended purpose/intended use* of the device and first it must be checked whether the intended use in the research/clinical investigation is within the devices’ intended purpose. The correct formulation of the intended use of NIBS devices is crucial for their regulatory approval.^5^

While the US FDA uses the term “intended use”, the MDR defines “intended purpose”.^6^ The MDR defines the “intended purpose” in Article 2(12) as follows: ‘*Intended purpose’ means the use for which a device is intended according to the data supplied by the manufacturer on the label, in the instructions for use or in promotional or sales materials or statements and as specified by the manufacturer in the clinical evaluation;*

The intended purpose is determined for a given device by the manufacturer based on clinical evidence. The intended purpose for patients describes the intended medical use and must be formulated in a clear and precise manner and must be justified by clinical data on safety and evidence. Of note, the ‘indication for use’ is of particular importance for researchers as it refers to the clinical condition that is to be diagnosed, prevented, monitored, treated, alleviated, compensated for, replaced, modified or controlled by the medical device. It should be distinguished from ‘intended purpose’, which describes the effect of a device.^7^

Besides the intended purpose, a device’s Technical Documentation should also be submitted to the regulatory authorities (the labeling of the medical device, instructions for use, and promotional or sales materials, etc).

### 2.3 Functions and responsibilities of researchers, clinicians, manufacturers, service providers and ‘sponsors’ (responsible for the regulatory, financial and ethical aspects of the study) during the research/clinical study

The MDR offers many options on how to handle research/clinical investigations. The sponsor is responsible for determining the appropriate regulatory pathway for their clinical/research investigation. If the sponsor is uncertain as to which pathway should apply to a particular investigation, the National Competent Authority should be consulted. If the research/clinical data are to be used to support conformity assessment (e.g., for companies), the clinical investigation will fall under Article 62 of the MDR, otherwise another regulatory pathway may be chosen (for more information consult the Article 82 of the MDR^8^).

To assess if the use of a medical device in a research/clinical investigation is within its intended purpose, the sponsor must review its instructions for use. The following documents should be or are suggested to be collected by the promoter (Principal Investigator, researcher, etc) of the research trial and submitted to the sponsor (research institutions, clinics, study centers) and ethical committees: i) The EU declaration of conformity; ii) The labeling supplied by the manufacturer; iii) the CE conformity certificate for the device; iv) the clinical evaluation report/protocol or at least the manual i.e. the instruction for use.

- If the stimulator is a medical device, CE-marked and will be used within its intended purpose, the provisions on vigilance laid down in Article 80(6) and Articles 87 to 90 of the MDR shall apply.
- If the stimulator is a medical device not CE-marked or is CE-marked but will be used outside its intended purpose, the provisions on regulatory and safety reporting laid down in Article 80 of the MDR shall apply (MDCG 2020-10/1 Rev 1).^9^ The duration of the regulatory process can be several months.

During the study, clinical investigational reports (including safety reports) must be regularly submitted to the National Competent Authorities by the sponsor (there might be slight differences depending on the EU member’s states’ requirements).

Clinical investigations that are currently being conducted with NIBS devices and started prior to 26th May 2021 under Directive 93/42/EC and Directive 90/385/EC can continue to be conducted. Nevertheless, SAEs and device deficiencies occurring after the date of application of the MDR, must be reported to the National Regulatory Agencies according to the rules defined in Article 80 of the MDR. With the critical lack of these agencies/manpower and other resources in the agencies to approve the MDR compliance, the EU prolonged the transition period until 2028.^10^

When submitting a new proposal for evaluation in most of the countries, the process is sequential (first local ethical committee after National Regulatory Authorities) or parallel. Besides the above mentioned documents (study proposal, information for research participants, consent forms, case report forms, the complete documentation of the medical device including preclinical tests, risk–benefit evaluations, safety documentations, technical documentations, CE certificates), the followings also should be submitted: CVs of the applicants; the list of previously performed studies with medical devices (publication lists are not accepted); Good Clinical Practice (GCP) and medical device regulation certificates; training certifications related to the medical devices supposed to be used in the planned study (please note, in many countries the main investigator can be only a medical doctor when multicenter studies are planned); financial disclosure statements, detailed description of the study place (labs, rooms, equipment), number of parallel running studies with the same or different indications; the responsibilities and rules of the sponsor and study place at the case of adverse and serious adverse effects; delegation list concerning the tasks and responsibilities; a written power of attorney if not the sponsor is the submitting person or organization.

After submission, the authorities must respond within a time window (10–20 days), they can reject the proposal, or they can ask for additional documents. In some cases, e.g. in Portugal, after two rejections the researchers cannot submit the same study again.

## 3 Stakeholders’ perspectives

In the following sections, we provide a summary of the perspectives of different stakeholders on the existing regulatory frame-work in the EU. The stakeholders were invited on the basis of their previous work and/or experience in the field of brain stimulation. Additionally, we present possible solutions to address the challenges that have been posed by this framework at different levels.

### 3.1 Researchers’ perspective

#### 3.1.1 Issues/problems

The barriers to basic and clinical research introduced by the recent changes in the regulatory framework for NIBS in the EU are substantial.

i) The lifetime of research projects (limited by its funding agencies) is insufficient to accommodate the additional bureaucratic processes that significantly delay research.
ii) The costs required to meet the new regulatory requirements cannot be accommodated by the limited funding of research projectsorthelimitedfinancialwealthofresearchinstitutions.

#### 3.1.2 Consequences

i) *Administrative impact:* Increased bureaucracy requires research institutions to expand research time in administrative processes and lengthy review processes by funding agencies, ethical committees and National Competent Authorities. This results in serious delays in research. Moreover, National Competent Authority registration/approval is frequently required to be completed by the time of submitting or approval of project applications to funding agencies. This means that for non-medical intended purposes (e.g. basic brain research) or for new intended medical use, additional time and resources must be planned for before applying for such funding.
ii) *Financial impact:* An application to a National Competent Authority/Ethical Committee for a study using a medical device involves fees (800–2.000 Euro, depending on the country), as well as any amendment to the protocol during the course of the study (200–500 Euro per change). If the NIBS protocol is not within the intended purpose (e.g., basic research in healthy subjects in order to test a new protocol), other fees may apply, namely insurance for study participants. There are also increased financial costs associated with the increased bureaucracy, which requires research institutions to either expand research time in administrative processes or to hire dedicated administrative staff. For example, in Germany, to comply with the regulatory process, a study center needs approximately 70.000 Euro to accommodate the new regulatory costs associated with the preparation of a small study (n = 60). There are countries, in which the submission fee can be waived when the research is not funded by the industry (e.g. Finland), while in other countries that is not an option. This affects the freedom of research too and may add inequality between research funded by the industry and research that is not.
iii) *Delay starting clinical research and intervention:* Currently, any new indication and application for clinical research use must be submitted to the National Competent Authorities, even for the slightest deviation in the use of the device or protocol. The variety of stimulation parameters is almost infinite and, in practice, it is very often necessary to adapt the stimulation protocol to the unique set of symptoms presented by a specific patient, using a treatment that is not yet officially recognized. In keeping with other advances in medicine, approaches are moving to tailor the right therapeutic strategy for the right person at the right time.^11^ Personalized stimulation is being developed and the new regulation may forbid adjustment of, e.g. electrode positions or current intensity at the individual level. At the same time, the EU seems to provide a legal framework to support personalized medicine, at least at the pharmaceutical level. However, in the NIBS field, as a result of the regulatory framework, clinical treatment is, and will continue to be, seriously hampered.

Many ethical reviewers are also now confused as to how they should view, for example, a Class IIa medical device, when it is used for a non-medical indication (basic research). Researchers and clinicians frequently need support from companies, in order to respond to the questions. These challenges are causing unwarranted delays and increasing the expenses associated with research and patient care, with no apparent safety benefits, based on current scientific knowledge.

iv) *Setting back research in NIBS within the EU region:* a byproduct that we foresee of these financial and administrative burdens in the future, will be a decreased enthusiasm by funding agencies for NIBS research, due to these additional burdens/costs, hindering research teams and institutions in the EU to pursue innovation progress. This means that NIBS research in the EU will fall behind and experience slower progress when compared to the pace of research in other parts of the globe. As parts of Asia, North America, UK and Australia do not have scientifically limitative regulations, researchers located in these areas will consequently have key advantages in patenting and publishing their scientific outcomes compared to researchers located in the EU. For example, the US FDA advised that all TMS devices for neuro-logic and psychiatric disorders are considered Class II devices.^12^ The US FDA uses an approach whereby the local ethics committees can determine whether a device or protocol is considered non-significant risk and bypass the need for an FDA investigational device exemption.^13^ Similarly, in Canada, if a medical device has been approved by Health Canada for a specific indication an investigative team can proceed with local ethical committee submission for basic neuro-science research, different clinical indications or novel stimulation parameters.^14^ The current EU framework will impede and already impedes the ability of the EU researchers to lead innovation and will discourage the EU as a location for international collaborative neuroscience efforts. This situation, besides jeopardizing the well-earned position of European researchers as key leaders in NIBS, will, in the long run, limit EU research participants and patients’ access to the research-based innovation. Consequently, the EU – in a self-defeating and incomprehensible manner – will no longer be competitive in this area.

#### 3.1.3 Potential remediation / possible solutions

i) Amendment of the Regulation to allow local ethical committees to regain their ability to approve NIBS studies without the involvement of the National Competent Authority as is the case in North America, with appropriate technical safety documentation, and as long as it is guaranteed that they will have appropriate expertise in evaluating these issues. In this context, a clearer separation between patient care and research would allow us to continue with scientific progress without unnecessary delays. Furthermore, the use of CE certified NIBS devices, even if they have a different intended purpose (e.g., stimulators with an intended purpose for neuropathic pain could be used in patients with other types of pain disorders, such as lower back pain and cancer pain if it can be shown that the risk profile remains unchanged) with the approval of local ethics committees would facilitate clinical research, which will in turn potentially expand the evidence-based usage of NIBS. Such a regulation might substantially reduce the workload of Institutional Review Boards (IRBs). Overarching ethical concerns in the unnecessary waste of administrative and research resources might be appropriate, based on previous experiences (Snooks et al., 2023).
ii) There should be a fast-track procedure through the National Competent Authority that allows medical devices to be used in research settings (including non-intended use) as long as their risks are properly managed in the clinical investigation process. Concerning non-medical use, Annex XVI of the MDR is clearly intended for “neuroenhancement” brain stimulation techniques such as consumer stimulation devices, which are marketed to enhance intelligence or working memory performance: *‘Equipment for transcranial electrical stimulation to enhance cognitive performance; – Equipment for transcranial magnetic or electromagnetic stimulation to enhance cognitive performance.’* https://health.ec.europa.eu/system/files/2023-12/mdcg_2023-5_en.pdf. According to Commission Implementing Regulation (EU) 2022/2346 laying down common specifications for the groups of products without an intended medical purpose listed in Annex XVI to Regulation (EU) 2017/745 on medical devices: ‘*in general it is not possible to demonstrate equivalence between a medical device and a product without an intended medical purpose where all available results of clinical investigations relate to medical devices only. Therefore, clinical investigations should be performed for products without an intended medical purpose*.’ https://health.ec.europa.eu/system/files/2023-12/mdcg_2023-6_en.pdf. We understand that policymakers want to limit the risks of unregulated neuroenhancement – something upon which we all agree. However, at present, well-established clinical and research applications have also been unnecessarily affected because regulators are more concerned about the potential abuse of these techniques and devices than enabling the clinical research with these devices and techniques. Facilitating regulations will ensure speedy and cost-efficient research projects, especially those that involve healthy human volunteers.

The Reclassification (C/2022/8638) was intended to close an unintentional gap in the MDR classification rules 9 and 10, which allowed devices without a medical purpose to avoid conformity assessment and to be placed on the market with less oversight. Devices that are used in clinical investigations are subject to additional controls through the clinical investigation requirements and the Class III Reclassification unnecessarily impedes clinical investigations without adding any level of safety for research participants. It is extremely difficult for a researcher to convince an ethics committee of the safety of a device when they are faced with sentences such as “*Such modifications can have long-lasting effects and any unintended effects may be difficult to reverse*”.

iii) An exemption for research devices under development would solve several of the problems. In research with new devices, the results often lead to adaptation of the devices. One model for all kinds of research does not seem to fit all. Furthermore, if the model has been developed for pharmaceutical treatments and drug development, this kind of development may be so different by its nature that new ways are needed. The current regulatory framework requires a complete re-certification of a device even for minor changes.
iv) Recent experience shows that the registration and approval process in many countries is lengthy. In addition, the staff at the National Competent Authorities often have limited or no experience with NIBS methods. Calling for experts to join Competent Authorities, enhancing training in NIBS and increasing staff numbers would help overcome the current bottleneck in the NIBS field within the EU area. However, on the other side, bringing down the regulations to a required minimum would minimize administration staff, speed up administrative decision times and oppose the automatism in administrative growth.

### 3.2 Clinicians’ perspective

#### 3.2.1 Issues/problems

Using medical devices that have been thoroughly tested for safety in clinical populations should be the standard. The introduction of novel parameters with approved medical devices that are technically safe and within or close to the limits of known biological safety ranges is also a means of improving clinical practice, reducing cost and increasing quality of care without compromising safety. By analogy with off-label use in pharmacology, where the safety of an authorized drug has already been demonstrated, off-label use of an approved NIBS device should be allowed for brain stimulation by following established pharmacological rules. NIBS is one of the few clinical applications in medicine where the clinician plays a pioneering role in determining the indication, the optimal target location and stimulation parameters, similar to the process, when doses of medications are individually adjusted. It could be argued that patient’s should have the right to have access to ‘off-label’ treatment with a medical device if the clinician believes that it is medically indicated and the patient has had the opportunity to have full informed consent.

#### 3.2.2 Consequences

i) *Undermining access to treatment*: In the medium term, the EU regulatory framework will also make these treatments less accessible to patients. In particular, the EU regulatory frame-work will impede access to new developments in the field of clinical research. This will directly worsen and increase the suffering of millions of patients in the EU who could benefit from the therapeutic effects of new NIBS protocols and indications. This is a dynamic field worldwide, with new protocols and indications being added very frequently. However, research participants in the EU will be denied or severely restricted to get novel treatments under the current regulatory framework. Such a setback is not only felt by the medical specialties that use NIBS technology, but also by other medical specialties that use medical devices and face similar problems.^15^
ii) *Additional costs in the clinic*: In the short term, additionally, the Reclassification leads to increased costs and significant delays in the development of NIBS protocols in the clinical practice and updates in Europe, further undermining the leading role of European clinical research and intervention.

#### 3.2.3 Potential remediation/possible solutions

Consideration should be given to the removal of unnecessary and unprecedented bureaucratic barriers that currently impede professional clinical judgment in the use of already approved NIBS devices for treatment. The use of already approved NIBS devices should be subject to the same approach as the use of approved/licensed medicines. Delaying the development of potentially beneficial treatments may result in unnecessary suffering for patients who could benefit from this form of treatment. Accelerated approval is needed for protocols that are optimizations or minor variations of already approved protocols.

### 3.3 Patients’ perspective

This section represents the opinion of patients and patient representatives from Portugal, the Netherlands and Belgium. In this patients’ group, several of the patients were referred to an institute specialized in NIBS by general practitioners and psychologists. Before the treatment, practitioners provided detailed information on the procedure and patients could make a conscious and well-informed decision to start therapy.

#### 3.3.1 Issues/problems

i) *Setting back the accessibility to novel treatments in clinical research*: Patients that benefit from the use of TMS and other forms of NIBS, such as tDCS, usually have a long history of dealing with psychiatric and/or neurological disorders and suffering from depressive thoughts, anxiety, panic attacks, chronic pain and other symptoms. These symptoms have significant negative effects on their daily functioning, well-being, sleep, relationships and mental health, some even on a daily basis. Although for the treatment of many conditions good pharmacological options exist, they may not be sufficiently efficient or may have contraindications or unwanted adverse effects. Therefore, introduction of new therapies, concepts or instruments instills patients with the hope for some relief in the near future. Often this hope is already tempered before the therapy is accessible for patients, because e.g. more scientific research has to be done, safety standards have to be developed, or the effects in the long term are not clear yet.
  In recent years, the accelerated progress in several medical technologies and their combination in research and development has widened the range of conditions that can be treated with NIBS devices. In addition, technological advances have made it possible to develop different types of new brain stimulation devices (smaller, easier to use devices, linked to mobile phones apps for use at home, while in contact with the responsible physician). As a result, more patients have become aware of and open to the possibility of undergoing treatment with TMS or low intensity tES. In this context, clinical trials have been important in determining the optimal target location (e.g., by using neuronavigation) and stimulation protocols for each indication and patient, reinforcing the importance of personalized therapeutic approaches. For example, in some cases, stimulation approaches might work best with different stimulation targets according to the uniqueness of symptoms of each individual or as a complement to pharmacotherapy, psychotherapy and physiotherapy.
ii) *Misleading information about safety of NIBS*. When patients decide to start a treatment with NIBS, they generally consider the following factors: safety, efficacy, accessibility (easy and quick access), and costs. The information that is taken into account is provided by a psychiatrist, psychologist, neurologist and/or a general practitioner (e.g., family doctor), often supplemented by information found through internet search engines. Safety issues are playing the most important role in the decision to enter into a therapy; the highest safety standards are also in the interest of the patients. Nevertheless, any medication available on the market comes with a list of potential adverse effects, long enough to deter the most positive-minded patient, but these medications still remain available. From a patients’ perspective it is difficult to understand the underlying motivation for the risks described in the Reclassification, as patient safety (as described by the MDR) is simply not at risk. This decision clearly does not improve the current and future position of EU patients in any way.
iii) *Ethical aspects, the problem related to the distinction between research and care*. The current regulatory framework rests on the unfounded evidence of (risks of) harm. Consequently, the wish to protect the patients and research participants fails in two respects. For patients, the failure means that due to the obstacles in gaining evidence, they are deprived of evidence-based treatment. This actually increases their vulnerability (analogous to the cases of children and pregnant women in medical research). For research participants, the failure results in not having access to experimental treatments when for instance, the evidence-based options have not brought about the expected and desired results. For both, the incorrect information is likely to build even more boundaries in accessing this kind of treatment when their concerns (that are based on the unfounded evidence of risks of harm) grow.

#### 3.3.2 Consequences

i) *Access to unclear information*: In terms of safety, the reclassification of NIBS devices to Class III (highest risk) may cause (or has already caused) some confusion and concern among the patients and their communities, and reduce their will-ingness to try this type of treatment or participate in clinical studies. The Reclassification also mentions a lot of adverse effects, which might deter patients. However, according to the personal experience of these patients and patients’ groups, none of these adverse effects ever occurred during their NIBS treatments. Patients should have access to clear, scientifically-based information about the safety and potential hazards of NIBS technologies, in order to make an informed decision about their therapeutic options. The current situation of lack of consensus and ambiguity may lead to confusion, questioning, lack of trust and ultimately rejection of NIBS treatment options.
ii) *Prolonged processes in evidence based treatment using personalized protocols*: A major advantage of NIBS treatments is that they can be timely and personalized, as slightly different stimulation protocols might be suitable for different patients/indications. With the new regulation, these small changes to established protocols will not automatically be accepted and will have to go through several lengthy processes. While this means more control over safety issues, which may be reassuring for some patients, the fact that each protocol has to go through a long process of registration and approval could delay treatment. As most patients need an immediate intervention, the time required for approval is not compatible with their needs. Therefore, this regulatory change means that some patients will not be able to receive a NIBS treatment on time due to bureaucratic constraints.
iii) *Accessibility should be taken into account*: Current regulatory changes could delay or even prevent access to NIBS treatment options, with negative consequences for patients. Higher standards and/or more bureaucratic procedures lead to higher costs and less availability of instruments to the patients. Higher costs also mean that health insurance companies will be less able and willing to (partially) finance the treatment of their customers. This new regulation does not consider patient accessibility to NIBS through appropriate reimbursement strategies. This is an important issue, as NIBS treatment options should be accessible to everyone.

#### 3.3.3 Potential remediation / possible solutions

To address the problems described above, the following actions may be useful:

i) Establish a clear position in the EU on the real safety and potential hazards of NIBS treatments, according to recently published scientific evidence. The justification for the classification as Class III / Class IIa devices should be based on scientific evidence and clearly explained in a way that is accessible to patients.
ii) Create a fast-track approval process for new treatment protocols that are optimizations or minor variations of already approved protocols and devices.
iii) Establish appropriate reimbursement strategies, so that all patients might have access to NIBS treatment options.

According to the conclusions of the patients, the Reclassification of NIBS-instruments, although it is presented as a regulation that is in the interest of the safety of patients, is introducing technical and bureaucratic burdens for the further development of NIBS-methods without taking the dissemination of reliable evidence-based information, accessibility to treatment and the well-being of patients, into account. The safety risks are not seen as realistic and the situation in essence does not differ very much from the health risks of readily prescribed medications. Of course, the stimulation should be done by trained professionals based on tested protocols. The patients must be enabled to take a well-informed decision on whether they want to enter therapy and what (realistically presented) risks are involved. In this sense, the Reclassification cannot be seen as an improvement of the position of patients in the EU.

### 3.4 Manufacturers’ perspective

#### 3.4.1 Issues/problems

On behalf of the EU-based tDCS manufacturers as well as TMS manufacturers outside of the EU with a substantial presence in the EU research and clinical fields, represented in this document, we express our serious concerns about the unforeseen negative consequences that the recent Reclassification of non-medical brain stimulation devices by EU regulators may have on research, innovation and clinical outcomes. We believe that a well-regulated market is essential for the growth of the industry by focusing on customer’s health or well-being, and we welcome regulation based on the principles of EU’s Better Regulation framework, where policymaking is evidence-based, legislation avoids unnecessary burdens and stakeholders are involved in the decision-making process (e.g. classification of devices). In our view, the introduction of reclassification has fallen short of these principles.

#### 3.4.2 Consequences

The lack of rigor and stakeholder involvement in the Reclassification has created a complicated situation for companies for a number of reasons.

i) *Inability for companies to react to the new regulatory frame-work*: The unclear situation until the announcement of the MDR declarations and guidance documents and their rapid timetable for implementation did not allow companies in cooperation with their Notified Bodies/Regulatory Agencies to react if their product was reclassified and subjected to additional and potentially costly controls. Until now, this process is still not fully understood. In case that manufacturers of Medical Devices need to reclassify too, only a limited number of Notified Bodies handle Class III devices, putting additional pressure on an already strained National Competent Authority system and resulting in unexpected delays for the manufacturers.
ii) *Increasing delay and costs*: For class III devices, a premarket clinical investigation is required. This requirement will contribute to higher expenses and delays in the process.^16^
iii) *Withdrawal from NIBS area*: Companies in our sector often hold the largest data sets on the real-world use of NIBS devices including post marketing data analysis that provides researchers, regulators and the public with insights into their safety and performance. Failure to follow the principles of Better Regulation, involving relevant stakeholders and taking into account the latest evidence, will force EU-based companies to consider focusing on more transparently regulated areas. As a result, the EU will not be at the forefront of innovation in this sector.
iv) *Decreased competitiveness*: In light of the lack of transparency of the MDR requirements, the additional costs and delays incurred, biomedical companies currently operating within the EU may contemplate reevaluating their presence. This, in turn, has the potential to impact competition within the EU, leading to reduced product quality, increased device prices, and, ultimately, a direct influence on patient well-being. A decrease in competition in the medical market could result in slower innovation, limited options for patients and healthcare providers, price inflation, lower quality control standards, and reduced accessibility to essential healthcare services, particularly for economically disadvantaged patients.

We believe that there was an inadequate hearing process for the Reclassification, given the magnitude of the first use of Article 51 (3)(b) of the MDR and the far-reaching implications of the inherent risk descriptions in Recital 7 of the Reclassification. We also believe that the EU Commission has not provided sufficient evidence, either scientific or vigilance, to justify the triggering of Article 51 (3)(b) or for the descriptions in Recital 7.

#### 3.4.3 Potential remediation/possible solutions

*Review of the current regulatory framework in the EU:* The correct solution would be to conduct the hearing in a correct manner, conduct a rigorous scientific review on safety, and then based on that review the need to reclassify the technologies in question. If the conclusion remains to reclassify, then the exact wording used in the Reclassification must follow the latest and best available evidence as uncovered in the scientific review, and clearly distinguish this from the medical intended purposes.

*Minimal solution:* If the EU Commission is unwilling to admit to the lack of rigor in the hearing process, and to object to the claim that the evidence provided is insufficient to trigger Article 51(3) (b), there is an alternative minimal solution that would address the specific problem related to the incorrect and unsubstantiated inherent risk descriptions in 2022/2347 Recital 7.^17^ To remedy this, the Commission would only need to revise the incorrect statements in Recital 7.

The Commission is further requested to provide clarity, as attempted in the newer guidance documents MDCG 2023-5 for NIBS devices to enhance cognitive performance, which other NIBS products fall under Annex XVI, if NIBS devices for research belong to it or not, and if a premarket clinical investigation might or might not be compulsory to bring Annex XVI NIBS products on the market and how the obtaining of preclinical data is regulated. This solution can be implemented within the existing frameworks for amending regulations, as recently demonstrated with Annex XVI amendment C/2023/3948.

## 4 Position of the European Society for Brain Stimulation (ESBS)

The European Society for Brain Stimulation (ESBS), founded in 2022, is an independent professional association of physicians, psychologists, neuroscientists, biologists and others, specializing in the research and clinical application of NIBS techniques. The ESBS has a wide pan-European membership including representatives of 16 countries. The mission of the ESBS is to represent and promote the field of NIBS research and clinical practice in Europe based on the latest scientific evidence.

The ESBS strongly opposes the Reclassification and the strengthening the MDR, which already has and will have significant negative consequences for the future of our field and our society. The Reclassification of NIBS devices with non-intended medical purpose as having the same level of risk profile *as invasive brain stimulation devices that are implanted inside the brain* is conceptually wrong and therefore inappropriate, contradicts 30 years of safety data, and has been decided without consultation of relevant experts. Based on the vast amount of safety data collected, *meta*-analyses, reviews, guidelines and consensus papers, the current scientific and clinical evidence suggests that both rTMS and low intensity tES are safe treatment and research interventions with few and mild AEs. This position is fully endorsed by the International Federation of Clinical Neurophysiology (see below).

The ESBS agrees that all NIBS devices must be certified as a medical device. Safety is our first priority. We advocate that a NIBS device should be classified according to the actual inherent risk associated with each device in question, taking into account the type, density and location of energy application (Class IIa). We are therefore strongly opposed to the Class III decision, and we urge our colleagues working in our field to do the same, regardless of their nationality. We have already sent a letter of protest to the EU. See also our website (https://www.brain-stimulation.eu/manifesto-eu-reclassification-of-nibs/ and Baeken et al., (2023)) for more details.

## 5 Position of the Europe-Middle East Africa Chapter of International Federation of Clinical Neurophysiology (EMEAC-IFCN)

The International Federation of Clinical Neurophysiology (IFCN), founded in 1947, is a federation of societies from four chapters, Europe, Middle East and Africa (EMEAC-IFCN), Asia-Oceania, Latin America, and North America, including around 9000 members. The mission of the IFCN is “*to* pr*omote the best practice in clinical neurophysiology through education and research throughout the world*”, and its vision is *“to improve healthcare worldwide by understanding the nervous system and optimizing the diagnosis and treatment of its disorders through clinical neuro-physiology*”. The EMEAC-IFCN chapter has some of the NIBS pioneers among its members.

Clinical neurophysiology thrives as a discipline if it continues to explore new ways of studying the bioelectric activity of the nervous system. We are concerned that an ongoing tightening of regulations on medical products will hinder the research in our field, and delay patient care. Therefore, the EMEAC-IFCN strongly opposes the European Reclassification of NIBS and the above-mentioned strengthening the MDR, which will have a substantial negative influence on the future of our field. Annex XVI of the MDR is obviously designed for non-medical “neuroenhancement,” like consumer stimulation devices. We need clear regulations, distinct from these consumer devices. We also need a clearer separation of patient care devices and research devices and a more flexible process for research with non-medical devices. During research, results often lead to adaptation of the devices to new requirements. The present situation requires a totally new certification of a device even for minor changes. Similar problems occur in the software area.

Whilst we understand and support the necessity of safety measures in the area of patient care and research, the vast amount of literature has provided evidence of the safety of NIBS. The EMEAC-IFCN agrees that all NIBS devices must be certified as medical devices, but we strongly disagree with the Class III decision. We have already sent a letter of protest to the EU, and published it on our website https://www.ifcn.info/docs/Feedback-Europe-Middle-East-Africa-Chapter-IFCN.pdf.

## 6 Position of the Clinical Transcranial Magnetic Stimulation Society (CTMSS)

The Clinical Transcranial Magnetic Stimulation Society (CTMSS) is a nonprofit international medical society dedicated to optimizing clinical practice, supporting research, and increasing access to high-quality, evidence-based TMS. Established in 2013, CTMSS has over 1000 members from more than 35 countries worldwide, including TMS clinicians with extensive clinical and research experience.

The CTMSS strongly disagrees with reclassifying TMS and tDCS devices to Class III devices, the category with the highest risk. This classification significantly impacts clinicians and researchers in our field and will ultimately significantly reduce patient access to safe and effective treatments using non-invasive brain stimulation devices. TMS and tDCS devices are non-invasive neuromodulation devices. They do not use incorporated diagnostic functions to determine patient management. When used under the guidance of a medical professional, both TMS and tDCS devices have proven safety records with very low risk for adverse outcomes (Antal, 2017, 2022; Bikson, 2018; Rossi, 2021). As of 2020, at least 20 million TMS treatments have been delivered worldwide in clinical settings (Carpenter and Phillip, 2020). Our society was unaware of this reclassification and the feedback period. Based on the limited feedback to the short public hearing, we suspect that other relevant groups and experts in our field were also uninformed.

Although the Clinical TMS Society is a separate and independent international medical society, we fully endorse the statement and position as published by the European Society for Brain Stimulation (ESBS).

## 7 General conclusions

In the short term, the MDR and the consequences related to the Reclassification of NIBS devices without an intended medical purpose, will lead to higher costs, an increased bureaucracy and significant delays in NIBS research and development, undermining Europe’s historic global leadership in this field. In the medium term, this EU decision will ultimately make NIBS treatment less accessible to patients in Europe and it will seriously hamper research, device development, and the search for new or more refined clinical protocols. European citizens will be disadvantaged, and there is a risk that other less effective and more invasive treatment approaches with serious adverse effects will be overused to compensate for the lack of availability of NIBS. In particular, this will affect not only the use of TMS or tES, but also other NIBS applications such as transcutaneous auricular Vagus Nerve Stimulation (taVNS) and Focal Ultrasound (FUS).

### 7.1 The way forward – a mistake to be corrected

The regulatory update can be amended and improved in order to reverse or mitigate the negative consequences outlined above, including the regulation in the MDR (intended/non-intended purpose). This would require a new action by the EU Commission. The main route would be via the Member States of the Commission (exactly the same route as the previous Reclassification). This would require i) a new position on how NIBS devices should be classified; and ii) a revision of the justification for the change in the light of the recent scientific literature that was not considered at the time of the previous act. This can be a targeted effort through the European Parliament, EU national ministries, the EU Commission, and the MDCG. In particular, the stakeholders involved in this document are ready to engage with the policy makers responsible for the drafting and adopting legislation, interpreting the MDR and those involved in the National Competent Authorities and to support them in the next steps towards an improved EU regulatory framework for NIBS, a regulatory framework that ensures patient safety, while simultaneously promoting access to treatment, innovation progress, and competitiveness within the EU region.

## Footnotes

1 https://ec.europa.eu/commission/presscorner/detail/en/qanda_23_24.

2 https://health.ec.europa.eu/medical-devices-sector/new-regulations/guidance-mdcg-endorsed-documents-and-other-guidance_en.

3 https://eur-lex.europa.eu/eli/reg_impl/2022/2347/oj.

4 https://ec.europa.eu/info/law/better-regulation/have-your-say/initiatives/12972-Medical-devices-reclassification-of-products-without-an-intended-medical-purpose_en.

5 European Medical Device Regulation (EU) 2017/745.

6 https://health.ec.europa.eu/system/files/2020-09/md_mdcg_2020_6_guidance_sufficient_clinical_evidence_en_0.pdf.

7 https://health.ec.europa.eu/system/files/2020-09/md_mdcg_2020_6_guidance_sufficient_clinical_evidence_en_0.pdf.

8 https://health.ec.europa.eu/system/files/2021-04/mdcg_2021-6_en_0.pdf.

9 https://health.ec.europa.eu/system/files/2022-11/md_mdcg_2020-10-1_guidance_safety_reporting_en.pdf.

10 https://ec.europa.eu/info/law/better-regulation/have-your-say/initiatives/13684-Extension-of-the-transition-period-for-medical-devices_en.

11 https://health.ec.europa.eu/medicinal-products/personalised-medicine_en.

12 Medical Devices; Neurological Devices; Classification of the Transcranial Magnetic Stimulation System for Neurological and Psychiatric Disorders and Conditions AGENCY: Food and Drug Administration, HHS Federal Register / Vol. 84, No. 50 / Thursday, March 14, 2019 / Rules and Regulations page9228, [Docket No. FDA–2019–N–0396];

13 https://www.fda.gov/downloads/RegulatoryInformation/Guidances/UCM126418.pdf.

14 Guidance Document Applications for Medical Device Investigational Testing Authorizations ISBN: 978-0-660-27058-6.

15 https://www.biomedeurope.org/news/2022/330-biomedical-alliance-in-europe-calls-for-urgent-measures-to-ensure-the-continuing-availability-of-essential-medical-devices.html.

16 https://health.ec.europa.eu/system/files/2021–10/mdcg_2021-24_en_0.pdf.

17 Article 1 (c) in REGULATION (EU) 2022/2347 as in https://eur-lex.europa.eu/legal-content/EN/TXT/PDF/?uri=CELEX:32022R2347.
